# Sibling Ill Health and Children's Educational Outcomes

**DOI:** 10.1111/josh.12887

**Published:** 2020-02-27

**Authors:** Cristian Bortes, Mattias Strandh, Karina Nilsson

**Affiliations:** ^1^ Umeå University, Department of Social Work, Faculty of Social Sciences Umeå SE‐901 87 Sweden; ^2^ Umeå University, Department of Social Work, Faculty of Social Sciences, Umeå SE‐901 87, Sweden; Karlstad University, Centre for Research on Child and Adolescent Mental Health Karlstad SE‐651 88 Sweden; ^3^ Umeå University, Department of Sociology, Faculty of Social Sciences Umeå SE‐901 87 Sweden

**Keywords:** siblings, child and adolescent health, education, academic achievement, register data

## Abstract

**BACKGROUND:**

The presence of health problems in a child is known to be negatively associated with later academic achievement, but less is known about the educational outcomes for siblings of children in poor health. The study investigated how having a sibling with health problems affects a healthy sibling's academic achievement.

**METHODS:**

We utilized medical and social microdata from Swedish administrative population registers. Our sample consisted of N = 115,106 individuals (51.3% boys) born in 1990 in Sweden. We compared children with ill siblings to children whose siblings did not have poor health. Siblings' hospital admissions and the academic achievements of the healthy sibling during their final year of compulsory education (at the age of 15‐16) were analyzed using linear and logistic regression in relation to individual health‐ and family‐related confounders.

**RESULTS:**

Sibling hospitalization was significantly associated with lower overall grade points (β = −10.73, p < .001) and an increased odds ratio (OR) of ineligibility for upper secondary education (OR = 1.42, 95% confidence interval = 1.31‐1.52, p < .001).

**CONCLUSIONS:**

School and health personnel should also consider the needs of healthy siblings during their work with children in poor health, because they too can be disadvantaged.

Poor health in childhood and adolescence is negatively related to academic achievement.^1^, ^2^, ^3^ According to European and national governing documents, all children are entitled to equal education, schools should strive to compensate for differences in students' backgrounds and needs, and particular consideration should be taken to the interests of each individual child.^4^, ^5^ Health services can ensure these rights and comply with these requirements by adapting treatments to facilitate education or by providing hospital schools.^6^ Schools can contribute by offering additional resources to children who need extra support to achieve their academic potential.^7^, ^8^

However, children are nested in families, and if one member of a family has health problems, there are likely to be spillover effects on the other members of the family. To understand the implications of ill‐health for student achievement in a wider sense, it is therefore necessary to determine how the academic achievement of healthy children is affected by having a sibling in ill health. While research on the psychosocial consequences for healthy siblings is plentiful,^9^, ^10^, ^11^, ^12^, ^13^, ^14^, ^15^, ^16^ we know much less about their educational outcomes. Since education is crucial for several outcomes later in life, this deserves more attention than it has so far received. Although a few studies have demonstrated the existence of spillover effects between siblings in relation to achievement,^17^, ^18^, ^19^ most related research has been based on small convenience samples, focuses mostly on siblings of children affected by cancer, and is predominantly based on data from Anglo‐Saxon countries. Consequently, prior research suffers from weak generalizability and we lack evidence on how the matter stands in a Scandinavian context. The purpose of the present study was therefore to investigate the relationship between having a sibling with health problems, not only cancer, that required repeated hospitalization and academic achievement, by using medical and social microdata from Swedish total‐population registers.

We considered 2 research questions: (1) Is having a sibling with ill health related to academic achievement in the final year of compulsory education? (2) Do parental resources, measured by parental level of education, moderate the relationship between having a sibling with ill health and academic achievement in the final year of compulsory education? These questions are addressed using ordinary least squares and logistic modeling, adjusting for individual birth and later life health status as well as family‐level covariates. By drawing on resource dilution theory^20^, ^21^ and research on stress transmission mechanisms,^22^, ^23^ we provide perspectives that may explain how having a sibling with ill health could affect a child's academic achievement. The present article is the first to specifically examine the relationship between sibling ill health and a global educational outcome such as final grades during compulsory education using large national datasets. As such, and by providing evidence from a non‐Anglo‐Saxon context, it is a novel contribution to both the sibling and school health research literature.

## Previous Research

Among the many literature reviews on how childhood illness affects the well‐being, family adjustment, and psychosocial functioning of the ill child's siblings,^9^, ^10^, ^11^, ^12^, ^13^, ^14^, ^15^, ^16^ only one focuses explicitly on studies that examine school‐related outcomes: Gan et al. 24 reviewed 28 studies on the school *experiences* of >1470 siblings of children with chronic illnesses, primarily cancer. Most of the studies were qualitative in their design and examined outcomes including academic functioning, psychological effects, peer relations, and school attendance. The authors concluded that “siblings experience negative effects in psychological, social and academic domains of school functioning” (p. 31). However they also noted that the conclusions of these studies “may not generalize well beyond the experiences of siblings of children diagnosed with cancer” (p. 30) because of the limitations of their samples. Additionally, academic achievement was not examined as an outcome in any of the reviewed studies. Regarding specific achievement outcomes, Fletcher et al.^19^ found that having a sibling with developmental disability or externalizing behavior is associated with lower math and language test scores. Similarly, Breining^17^ found a negative influence of ADHD on siblings' school outcomes in ninth grade. Except for cases of positive spillover effects between siblings on achievement in families where one child has *special educational needs* 25—which does not necessarily imply a health problem, unlike in the case where a sibling suffers from a disease such as cancer—the pattern of findings suggests a negative relationship between having a sibling with ill health and the healthy child's academic achievement.

## Why Would Having a Sibling With Ill Health Affect Academic Achievement?

Having a sibling with ill health can be considered to affect academic achievement through 2 main mechanisms. The first stems from how the sibling's ill health might affect the availability of familial resources. Because siblings typically grow up in the same household raised by the same parents, they share limited parental resources. According to resource dilution theory,^20^, ^21^ these resources can be categorized as (1) parent‐child companionship, (2) parental attention devoted to the child, and (3) access to material goods (quiet study rooms, books and newspapers, computers, etc.). All 3 resource types are considered to have important effects on early life development and achievements in adulthood. While the concept of resource dilution was introduced to explain the effects of additional children in the family, sibling ill health may have a similar effect. Black et al.^18^ show that sibling spillovers are partly due to constraints on the parents' time and financial resources. Self‐reported data indicate that parents spend considerable time caring for children with poor health—on medical visits, home‐based therapies, and providing parental care—so healthy siblings receive less parental attention.^26^, ^27^ Ill siblings may also require costly therapies and their needs may reduce the parents' labor market participation and income, both of which would reduce the financial resources available to the healthy children. While family economic resources may be important in Sweden, they are, however, likely to be less important than in countries without comprehensive health and income insurance. Other limited resources such as time, attention and emotional support are more likely to be affected.

The second mechanism relates to the impact of the sibling's ill health on the child's own resources. Having a sibling with health problems may impose psychological costs on a child. These can be considered to result from stress transmission mechanisms whereby the sibling's ill health acts as a stressor for other family members and has negative effects on their well‐being. While some positive psychological outcomes have been reported, such as a stronger sense of maturity and independence, the literature generally indicates that having a sibling in ill health limits a healthy child's psychological resources,^14^, ^15^, ^16^ which may manifest as difficulty concentrating in school and completing homework,^28^, ^29^ or poor school attendance due to commitments relating to the ill child.^30^

In summary, although previous studies linking sibling ill health to academic achievement *per se* are scarce, it is reasonable to assume that the reported negative psychosocial experiences of children with seriously ill siblings^9^, ^10^, ^11^, ^12^, ^13^, ^14^, ^15^, ^16^ will also create problems in the school context and reduce achievement. Furthermore, although stress transmission mechanisms can be considered universal, resource dilution may be national context dependent.^31^, ^32^ In the Swedish context, as cross‐national comparative research has shown,^33^ the effect of potential resource dilution in the family on academic achievement is reduced by the country's institutional welfare system. Therefore, in reconnecting with our research questions, we expected to find a negative association between having had one or more siblings with ill health and academic achievement in the final year of compulsory education. However, parental education, as a proxy for family resources, was not expected to moderate this relationship.

## METHODS

This study was based on data from several national registers obtained via the Umeå SIMSAM Lab data infrastructure.^34^ The analyzed data include micro‐level medical and social information, and each individual is assigned a unique, fully anonymized, personal identification number (PIN) that links them to their parents and siblings across registers. The registers and variables considered in the study are listed in Table 1.

**Table 1:** List of National Registers and Variables Used in the Study

National Register	Variables	Categories
Multi‐Generational Register	Parental and sibling PIN	
Total Population Register	Parental country of birth	Nordic, non‐Nordic
	Family structure, married/cohabiting with both biological parents at age 16	Yes/no
Longitudinal Integration Database for Health Insurance and Labor Market Studies (LISA)	Parent's level of education	Compulsory, 2‐year secondary, 3‐year secondary, post‐secondary
The Medical Birth Register	Sex	Male/female
	Small for gestational age	No/yes
	Large for gestational age	No/yes
	Malformed child	No/yes
Apgar‐score at 5 minutes	Normal/low
National Patient Register	Sibling hospitalized during index‐person's age 0‐6 and 7‐12 and 13‐16	No/yes
	Index‐person hospitalization, age 13‐16	No/yes
Prescribed Drug Register	Index‐person received drug prescription for psycholeptics or psychoanaleptics school year 9	No/yes
Swedish National Agency of Education's Pupil Register	Overall grade points	Continuous 0‐320 points
	Eligibility upper secondary education	Yes/no

### Participants

The study population consisted of the cohort of individuals born in 1990 in Sweden, who were alive and residing in Sweden 2006, the last year of compulsory school for the cohort. Foreign‐born individuals were excluded because we wanted to account for health status at birth in our analyses. We also excluded singletons and compared children with ill siblings to children whose siblings did not have any indications of poor health. Our final analytical sample consisted of N = 115,106 individuals (51.3% boys), referred to below as index‐persons. Both full and half‐siblings (maternal and paternal) of the index‐persons are regarded as siblings.

### Dependent Variables

Academic achievement was measured using 2 variables: (1) *overall grade points* and (2) *eligibility for upper secondary education*. Overall grade points reveal differences in levels of academic achievement, while eligibility for continuation to upper secondary education reveals problems with successfully completing compulsory education.

Overall grade points is the sum of the 16 best subject grades in the 9th and final grade of compulsory school, which are received at the age of 15‐16. For each subject, a student is assigned a grade ranging from 0 to 20, where 0 indicates failure. The overall grade points thus range from 0 to 320 and indicate the child's general academic achievement; an overall grade points value of 0 denotes failure in all tested subjects, while an overall grade points value of 320 indicates that the child achieved the highest possible grade in all 16 subjects. Overall grade points is a continuous variable with an approximately normal distribution (M = 205.5 SD = 64.7).

Eligibility for upper secondary education was assessed when the cohort was in the ninth grade. It was assessed based on successful completion of compulsory schooling with passing grades in the core subjects of Swedish, English, and mathematics, which were required for admittance to national upper secondary level education programs. A binary variable was defined, with a score of 0 assigned for passing grades in all 3 core subjects (and thus eligibility for upper secondary education) and a score of 1 for failing one or more of the 3 core subjects (and thus non‐eligibility for upper secondary education).

### Independent Variables

#### *Sibling ill health*

Because hospitalization results exclusively from detrimental accidents or severe health problems, we defined children having a sibling with ill health as those children who had at least one repeatedly hospitalized sibling. Specifically, sibling ill health is a dichotomous (no/yes) variable taking a value of “yes” only if the index person had one or more siblings who had at least 3 (or more) separate overnight hospitalization events during each of the following 3 periods of the index person's life, namely the years between the ages of 0 and 6, 7 and 12, and 13 and 16. This operationalization captures siblings of children with a broad range of different and recurring health problems.

#### *Parents' level of education*

The parents' level of education is often used as an indicator of their socioeconomic status and indirectly influences children's academic achievement because it affects the parents' educational expectations and parenting behaviors. Higher educated parent's also spend more time with their children.^35^, ^36^ This variable is thus suitable to use for testing whether parental time, attention and material resources moderate the relationship between sibling ill health and academic achievement. We therefore used it to measure family resources. It was operationalized as the highest level of education attained by either parent when the child was 7 years old (the age at which most Swedish children start compulsory school), and indicates the parental resources available to the child throughout their schooling. Four levels were defined: compulsory education (reference), 2 years of upper secondary education, 3 years of upper secondary education, and post‐secondary education.

#### *Family structure*

The complexities, ambiguities, and stresses associated with new familial roles and relationships consume family resources that could otherwise be directed to different ends. Therefore, we included “family structure” in our analysis using a dichotomous variable indicating whether the child's biological parents were or were not (yes/no) married/cohabiting when the child received their final compulsory school grades.

#### *Parents born in a Scandinavian country*

Dichotomous variables were defined for the child's mother and the child's father, indicating whether or not they had been born in a Scandinavian country (Sweden, Denmark, Norway, Finland, Iceland) or a non‐Scandinavian country (yes/no).

#### *Index person's birth health*

To control for selection into poor health from birth, we included variables indicating whether the child was *small for their gestational age* (no/yes), *large for their gestational age* (no/yes), *malformed* (no/yes), and their *Apgar score 5 minutes after birth*, which quantifies the newborn's physical condition 5 minutes after birth (normal/low). An Apgar score below 7 is considered low, while a score of 7‐10 is considered normal.^37^

#### *Index person's later health*

Two dichotomous variables were used to characterize the health of index children later in life. The first, *psychotropic drug prescription*, indicates whether the child has ever been prescribed psycholeptic (for treatment of psychological disorders, bipolar disorder, anxiety, and insomnia) or psychoanaleptic (for treatment of depression and attention deficit hyperactivity disorder) medication during their last year of lower secondary education (no/yes), according to the Swedish Prescribed Drug Register. The second such variable, *hospitalization*, indicates whether the index child had or had not been hospitalized for at least one night during their lower secondary education/early adolescence, between the ages of 13 and 16 (no/yes).

### Data Analysis

The multiple linear regression procedure was used to assess how having had a sibling with ill health was associated with *overall grade points*. Independent variables were added stepwise to assess potential confounding; this was done by running 4 models separately. Model 1 included only the *sibling ill health* variable. Model 2 also included the family‐level control variables (*parents' level of education*, *family structure*, and *parents born in a Nordic country*). Model 3 included the variables from model 2 together with the variables relating to the index child's birth health (*small for gestational age*, *large for gestational age*, *malformed*, and *Apgar score at 5 minutes*) and later life health (*psychotropic drug prescription* and *hospitalization*). Finally, the fourth model included an analysis of the interaction between having had a sibling with ill health and parental level of education: 3 interaction terms were computed, one for each level of parental education defined above (excluding compulsory education, which served as the reference category). We also performed analyses featuring 7 levels of parental education but found no significant differences between the 2 operationalizations. The same steps were taken to determine how sibling ill health was associated with *eligibility for upper secondary education*. However, due to the binary nature of that variable, logistic regression was used.

## RESULTS

A total of 6945 children (6%) had one or more siblings with ill health according to the study's operationalization. Table 2 presents the study variables related to educational outcomes. In total, 2973 children (2.6% of the study population) received schooling in a special education facility or dropped out of school before ninth grade. These observations were not included in any of the models.

**Table 2:** Educational Outcomes Related to Study Variables

	Overall Grade Points, M (SD)	Ineligible for Upper Secondary Education, N (%)
All individuals	205.5 (64.7)	12,284 (10.8)
Boys	194.6 (62.2)	6971 (6.1)
Girls	216.9 (65.3)	5313 (4.6)
Mother's country of birth		
Nordic	206.1 (64.2)	9549 (8.3)
Non‐Nordic	201.2 (69.4)	2735 (1.4)
Father's country of birth		
Nordic	197.0 (70.3)	7455 (6.5)
Non‐Nordic	180.2 (73.4)	2832 (2.5)
Parent's level of education		
Compulsory	164.1 (69.6)	2390 (2.1)
2‐year secondary	186.6 (61.7)	6377 (5.5)
3‐year secondary	209.1 (58.5)	1279 (1.2)
Post‐secondary	230.6 (58.8)	2104 (1.8)
Family structure		
Married/cohabiting	213.1 (60.6)	6960 (6.0)
Not married/cohabiting	187.3 (70.2)	5322 (4.6)
Individual birth health		
Small for gestational age	194.2 (67.7)	421 (0.4)
Large for gestational age	203.4 (66.2)	393 (0.3)
Malformed child	202.2 (65.5)	422 (0.4)
Low Apgar 5‐score	200.6 (65.2)	103 (0.1)
Individual later life health		
Hospitalized age 13‐16	193.8 (70.6)	718 (0.6)
Psychotropic drug pres.	148.4 (83.2)	813 (0.7)
Sibling hospitalized	189.2 (69.8)	1099 (1.0)
No sibling hospitalized	206.5 (64.2)	11,185 (9.7)

Table 3 presents the results of the multiple linear regression analyses using *overall grade points* as the outcome. The coefficients in the table indicate how many more or fewer grade points (as a measure of achievement) a particular variable in the model is associated with. Model 1 shows that having had one or more siblings hospitalized for at least one night during 3 age periods (hereafter referred to as “sibling ill health”) was significantly associated with lower overall grade points (β = −17.29, p < .001), corresponding to a 8.4% deviation (Gaussian distribution) from the sample mean (M = 205.5). Model 2 shows that adjusting for gender and parental resources/characteristics attenuates this association (β = −11.10, p < .001), resulting in a 5.4% deviation in overall grade points from the sample mean. In model 3, which is also adjusted for individual birth health and later health, the association was only marginally further attenuated (β = −10.73, p < .001), giving a 5.2% deviation in overall grade points from the sample mean. In model 4 we introduced the interaction terms “sibling ill health × parent's level of education.” No significant interaction effects between having had a sibling with ill health and parental level of education were found.

**Table 3:** Linear Regression Models Showing the Relationship between Sibling Ill Health, Sociodemographic Variables and Overall Grade Points (Unstandardized Beta Coefficients, Standard Error in Parentheses)

	Model 1	Model 2	Model 3	Model 4
No sibling hospitalized	Ref.	Ref.	Ref.	Ref.
Sibling hospitalized	−17.29 (.82)***	−11.10 (.75)***	−10.73 (.36)***	−14.57 (2.30)***
Parent's level of education				
Compulsory		Ref.	Ref.	
2‐year secondary		17.31 (.72)***	17.76 (.73)***	
3‐year secondary		39.10 (.81)***	39.18 (.82)***	
Post‐secondary		59.40 (.72)***	59.63 (.73)***	
Family structure				
Married/cohabiting		Ref.	Ref.	
Not married/cohabiting		−21.20 (.39)***	−20.42 (.40)***	
Mother born in a Scandinavian country		Ref.	Ref.	
Non‐Scandinavian country		2.56 (.74)*	1.90 (.86)*	
Father born in a Scandinavian country		Ref.	Ref.	
Non‐Scandinavian country		−4.47 (.75)***	−4.15 (.80)***	
Index person's birth health status				
Small for gestational age (ref: no)			−6.88 (1.16)***	
Large for gestational age (ref: no)			−1.58 (.01)	
Malformed child (ref: no)			−3.06 (1.02)*	
Apgar 5 minutes (ref: normal)			3.86 (2.12)	
Index person's later health				
Psychotropic drug pres. (ref: no)			−54.58 (1.15)***	
Hospitalization age 13‐16 (ref: no)			−10.33 (.83)***	
Sibling ill health × parent's education				
2‐year secondary				4.00 (2.56)
3‐year secondary				3.65 (3.13)
Post‐secondary				5.01 (2.67)
Constant	206.48 (.20)***	166.00 (.71)***	167.30 (.75)***	167.65 (.75)***
N	112,133	111,725	106,778	106,778
r^2^	0.004	0.174	0.194	0.194

* p < .05.

** p < .01.

*** p < .001.

r^2^ = Adjusted R Square. Models 2 and 3 are adjusted for the index person's sex. Model 4 is adjusted for all the covariates. Dependent variable: overall grade points (range: 0‐320, M = 205.5, SD = 64.7).

Table 4 presents the results of the logistic regression analyses using *eligibility for upper secondary education* as the outcome. The first unadjusted model shows that sibling ill health significantly increased the odds ratio (OR) of ineligibility for upper secondary education (OR = 1.68, 95% confidence interval [CI] = 1.57‐1.80, p < .001). Both model 2 and model 3 show that despite being somewhat reduced after adjustments, sibling ill health still significantly increased the odds of ineligibility for upper secondary education (OR = 1.42, 95% CI = 1.32‐1.53, p < .001), (OR = 1.42, 95% CI = 1.31‐1.52, p < .001). In model 4 we added the interaction terms “sibling ill health × parents' level of education,” which revealed a non‐significant interaction between having had an ill sibling and the parents' level of education. Overall, the patterns in results for the 2 outcome measures were similar.

**Table 4:** Odds Ratios (ORs) of Ineligibility for Upper Secondary Education (Confidence Intervals in Parentheses)

	Model 1	Model 2	Model 3	Model 4
No sibling hospitalized	1.00 (ref.)	1.00 (ref.)	1.00 (ref.)	1.00 (ref.)
Sibling hospitalized	1.68 (1.57‐1.80)***	1.42 (1.32‐1.53)***	1.42 (1.31‐1.52)***	1.40 (1.18‐1.67)***
Parent's level of education				
Compulsory		1.00 (ref.)	1.00 (ref.)	
2‐year secondary		0.55 (0.52‐0.59)***	0.55 (0.52‐0.59)***	
3‐year secondary		0.27 (0.25‐0.30)***	0.28 (0.25‐0.30)***	
Post‐secondary		0.16 (0.16‐0.18)***	0.16 (0.15‐0.18)***	
Family structure				
Married/cohabiting		1.00 (ref.)	1.00 (ref.)	
Not married/cohabiting		1.75 (1.69‐1.83)***	1.73 (1.61‐1.80)***	
Mother born in a Scandinavian country		1.00 (ref.)	1.00 (ref.)	
Non‐Scandinavian country		1.82 (1.70‐1.95)***	2.14 (1.98‐2.31)***	
Father born in a Scandinavian country		1.00 (ref.)	1.00 (ref.)	
Non‐Scandinavian country		2.27 (2.13‐2.43)***	2.08 (1.93‐2.24)***	
Index person's birth health				
Small for gestational age (ref: no)			1.38 (1.23‐1.55)***	
Large for gestational age (ref: no)			1.09 (0.97‐1.22)	
Malformed child (ref: no)			1.20 (1.08‐1.34)**	
Apgar 5 minutes (ref: normal)			1.20 (0.96‐1.51)	
Index person's later health				
Psychotropic drug pres. (ref: no)			3.73 (3.40‐4.08)***	
Hospitalization age 13‐16 (ref: no)			1.27 (1.16‐1.38)***	
Sibling ill health × parent's education				
2‐year secondary				0.98 (0.80‐1.20)
3‐year secondary				1.06 (0.80‐1.41)
Post‐secondary				1.08 (0.84‐1.38)
Constant	0.12***	0.21***	0.19***	0.19***
N	112,133	111,725	106,778	106,778
Pseudo R2	0.002	0.101	0.112	0.112

** p < .01.

*** p < .001.

Models 2 and 3 are adjusted for the index person's sex. Model 4 is adjusted for all the covariates.

## DISCUSSION

Our findings show that having a sibling with poor health is significantly associated with lower academic achievement in the final year of compulsory school in Sweden. Whereas previous research has concluded that siblings of ill children have negative experiences of school,^24^ the present study expands on these findings by demonstrating that the negative experiences are reflected in lower grades.

We proposed 2 main mechanisms to explain how having a sibling in ill health might affect academic achievement. The first is based on an extension of the concept of resource dilution, which is generally hypothesized to be a function of sibling group size. We suggested that an ill sibling may consume more parental resources than a healthy sibling by prompting the parents to reallocate their time, attention and emotional support. As such, sibling illness would restrict a healthy sibling's access to these parental resources. As demonstrated previously, however, the extent of resource dilution due to sibling group size and child well‐being varies between national contexts.^32^, ^33^, ^34^ In the context of the Swedish welfare state—a comprehensive institutional system with universal healthcare free of charge (tax‐funded) and an educational system oriented towards inclusion and equality—a child's wellbeing is not solely dependent on resources provided by the parents. In this study we used parental level of education as a proxy for resources. Although the relationship between sibling illness and the healthy sibling's academic achievement was not moderated by parental level of education, the main effect was significant. Thus, one interpretation of our results is that the effect of having a sibling with ill health on academic achievement operates mainly through individual stress transmission mechanisms. Although highly educated parents spend more basic, educational and recreational *time* with their children,^35^, ^36^ this is an inconclusive interpretation. Future research using more accurate measures of parental time, attention, and support is needed to disentangle these mechanisms.

An advantage of using multiple sources of linked data from total population registers is that one obtains a very large sample size. Consequently, our findings can be generalized to a very large proportion of the Swedish population. The design of comparing children with ill siblings to children whose siblings did not have any indications of poor health provides support for causal inferences. Another strength of the study was the ability to control for many, although far from all, of the potentially relevant confounders. Because we adjusted for both individual birth health *and* later life health, we can infer that the effect of sibling ill health on school grades is significant.

### Limitations

While there are many strengths to this study, it has some limitations that should be noted. The use of hospitalizations to identify ill siblings captures individuals that specifically required in‐hospital care, and would thus be a reliable indicator of severe health‐related problems. However, it might not capture some illnesses or other disabilities/impairments that can be debilitating without requiring hospitalization. Our results may thus underestimate the impact of sibling illness on academic achievement but are sufficient to reveal a general effect. Furthermore, differentiating the cause of the hospitalizations by diagnosis would have revealed condition‐specific effects. This was not feasible due to data limitations.

### IMPLICATIONS FOR SCHOOL HEALTH

Our results showed that children with ill siblings achieve lower school grades as compared to children whose siblings did not have poor health. The study lends evidence to the existence of negative health spillover effects between siblings on academic achievement. Some practical implications can follow from these results. Because a student's final grades in compulsory education determine their eligibility for admission to higher education in Sweden, it is important for support services to acknowledge the adverse effects of sibling illness on adolescents' educational trajectories and future opportunities. Existing school support services primarily focus on the disadvantages facing children in poor health. However, our results indicate that school psychologists, counselors and teachers should also consider the needs of healthy siblings during their work with children in poor health, because they too can be disadvantaged. School‐based support services should adhere to a family‐centered approach which is inclusive of also the healthy sibling. A sibling support model in which the school and the parents share responsibility for stabilizing the sibling's experience and providing consistent support, as also suggested elsewhere,^38^ may be needed to alleviate these negative effects. Examples of actions that schools can take are:

- Psychoeducational support to the sibling and the entire school regarding the ill child. This can include individual counseling, but could also be implemented as a feature of theme days on health and well‐being, in which parents can be involved to share experiences. This can improve the knowledge and attitudes of students and school personnel as well as sibling school functioning.
- Increase teacher and school personnel involvement in order to identify signs of maladaptive coping in siblings. This can also facilitate student‐teacher bonding and sibling school engagement which in turn could enhance school performance.
- In addition to receiving psychological support at school through a counselor, specific sibling support measures that schools can take are flexible assessment deadlines or offering extra orientation days to reduce sibling's anxiety in the event of the ill child's disease intensification.

### Human Subjects Approval Statement

The Regional Ethical Vetting Board in Umeå approved all research based on data from the Umeå SIMSAM Lab, including the present study (Dnr.2010‐157‐31).

### Conflict of Interest

All authors of this article declare they have no conflicts of interest.
